# CaNDis: a web server for investigation of causal relationships between diseases, drugs and drug targets

**DOI:** 10.1093/bioinformatics/btaa762

**Published:** 2020-09-01

**Authors:** Blaž Škrlj, Nika Eržen, Nada Lavrač, Tanja Kunej, Janez Konc

**Affiliations:** Department of Knowledge Technologies, Jožef Stefan Institute, Slovenia; Jožef Stefan International Postgraduate School, Slovenia; Department of Knowledge Technologies, Jožef Stefan Institute, Slovenia; Department of Knowledge Technologies, Jožef Stefan Institute, Slovenia; Jožef Stefan International Postgraduate School, Slovenia; Department of Animal Science, Biotechnical Faculty, University of Ljubljana, Slovenia; Theory Department, National Institute of Chemistry, SI-1000 Ljubljana, Slovenia

## Abstract

**Motivation:**

Causal biological interaction networks represent cellular regulatory pathways. Their fusion with other biological data enables insights into disease mechanisms and novel opportunities for drug discovery.

**Results:**

We developed Causal Network of Diseases (CaNDis), a web server for the exploration of a human causal interaction network, which we expanded with data on diseases and FDA-approved drugs, on the basis of which we constructed a disease–disease network in which the links represent the similarity between diseases. We show how CaNDis can be used to identify candidate genes with known and novel roles in disease co-occurrence and drug–drug interactions.

**Availabilityand implementation:**

CaNDis is freely available to academic users at http://candis.ijs.si and http://candis.insilab.org.

**Supplementary information:**

Supplementary data are available at *Bioinformatics* online.

## 1 Introduction

Protein–protein and protein–drug interaction networks are becoming increasingly important for drug development (Ashburn *et al.*, 2004). Such biological interactions, whether predicted or experimental, are stored in various databases (Konc *et al.*, 2017; Szklarczyk *et al.*, 2019). However, the causal relationships between the interaction partners remain relatively unexplored. A causal relationship occurs when e.g. a transcription factor increases the expression of a particular protein. Knowledge of these relationships could open up new avenues for drug development and improve our understanding of the disease processes.

Tools to explore networks of causal relationships are available (Boué *et al.*, 2015; Licata *et al.*, 2020); however, the fusion of these networks with the knowledge about diseases and drugs into heterogeneous causal networks, should enable even higher understanding of diseases as well as more efficient drug discovery. In particular, such networks could be used for the study of disease co-occurrence, the simultaneous presence of two or more diseases in a patient (Hu *et al.*, 2016).

We developed Causal Network of Diseases (CaNDis), a web server that enables visual exploration of a causal network of protein–protein interactions enriched with annotations on diseases and drugs. We also constructed a new disease–disease network. Here, each node is a subnetwork in the original causal network, in which all the nodes are linked to the same disease. A score is assigned to a link between a pair of diseases based on the connectivity of the two subnetworks, representing disease similarity. CaNDis is an intuitive web server based on a fast WebGL viewer (see sections ‘Software aspects and scaling’ and ‘Comparison with other tools’ in Supplementary Information). It facilitates drug discovery with respect to the identification of disease co-occurrence and drug–drug interactions at the level of drugs, genes, proteins and diseases.

## 2 The CaNDis web server

The CaNDis heterogeneous causal network is based on the CBN (Boué *et al.*, 2015) and SIGNOR 2.0 (Licata *et al.*, 2020) causal biological networks. It is extended with protein–RNA interactions from the PRD database (Fujimori *et al.*, 2012) and drug–gene interactions from the DGIdb database (Cotto *et al.*, 2018); gene nodes are superimposed with the gene–disease annotations from the DisGeNET database (Piñero *et al.*, 2020). Causal pathways are merged into a single network containing the interacting proteins as nodes and the causal relationships, such as increase or decrease in expression levels, as links (see section ‘The CaNDis network’ in Supplementary Information).

Gene–disease annotations are added as attributes to the protein nodes and drugs and RNA are added as new nodes, which are linked to their respective target protein nodes. The obtained heterogeneous causal network contains annotations on more than 3000 complex diseases as well as on the FDA approved drugs to enable the investigation of diseases (i.e. co-occurrence of diseases) and drug–drug interactions (see section ‘Use in drug–drug interactions’ in Supplementary Information).

A new disease–disease network is also developed from the heterogeneous causal network (sections ‘Disease–disease network construction’ and ‘Normalized disease–disease similarity score’ in Supplementary Information), and is available on the web server.

## 3 Use for identification of genes with roles in disease co-occurrence

**Fig. 1.**
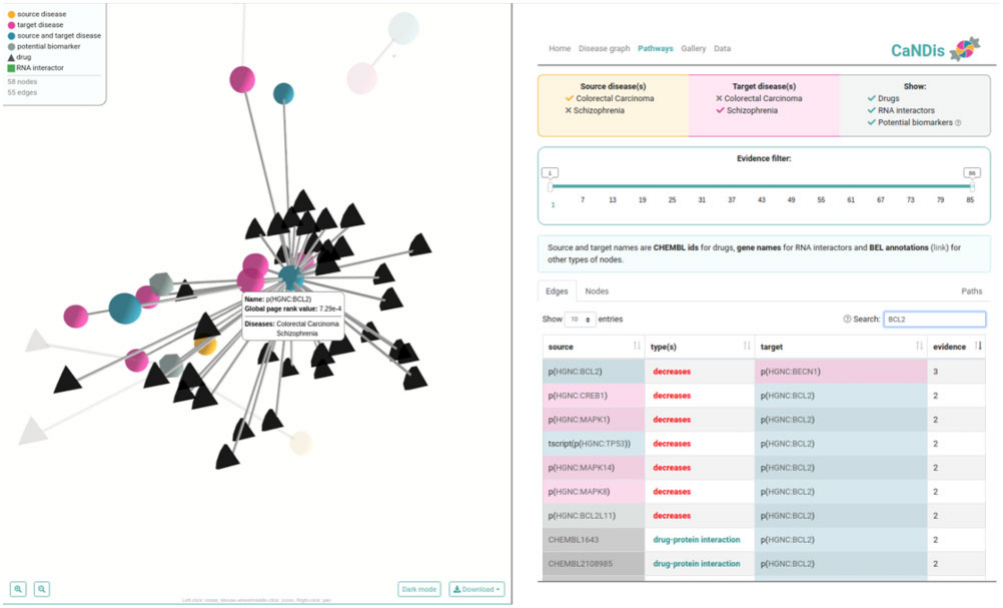
CaNDis web server causal interaction network for the *BCL2* gene. Left: proteins (identified by their gene names) associated with schizophrenia (yellow circles), cancer (purple circle), both diseases (blue circles) or none of the diseases (grey circles) represented as a 3D interaction network. Drugs are black triangles. Highlighted are the *BCL2* gene in the middle and its neighbors. Right: tabular view of the same interactions labeled with causal relationships (e.g. decreases). Some of the relevant neighbors of *BCL2* gene are *DDIT3, MAPK8, MAPK14, MAPK1, CREB1, PRKCA, CASP3* and *BECN1* (all genes are available on the web server)

According to epidemiological studies, patients with central nervous system (CNS) disorders, that is, Alzheimer’s disease, Parkinson’s disease and schizophrenia, are less likely to develop certain cancers (colorectal, lung and prostate), and vice versa. This inverse co-occurrence is attributed to a set of proteins, including the proteins coded by the *TP53* and *BCL2* genes (Behrens *et al.*, 2009; Ibáñez *et al.*, 2014; Tabarés-Seisdedos *et al.*, 2009), both of which are down-regulated in cancers and up-regulated in CNS disorders. Using ‘colorectal carcinoma’ and ‘schizophrenia’ as the inputs to the CaNDis web server, we successfully identified the *TP53* tumor suppressor gene and the *BCL2* anti-apoptotic gene as those in common to both the diseases (*BCL2* network is shown inFig. 1). In addition, we also found proteins that have not yet been associated with either disease (Piñero *et al.*, 2020), but which could play a role in this inverse disease co-occurrence due to their interactions with the protein coded by the *BCL2* gene (the list of proteins is in the caption of Fig. 1). Literature search showed that some of the identified proteins are indeed associated with both diseases. For example, the oncoprotein coded by the *DDIT3* gene (Engström *et al.*, 2006) is associated with lung cancer (Li *et al.*, 2015) and upregulated in schizophrenia (Umeda-Yano *et al.*, 2013). Similarly, *MAPK8* plays a role in both colorectal cancer and schizophrenia (Chandrasekaran *et al.*, 2012; He *et al.*, 2012; Szatkiewicz *et al.*, 2014). This illustrates the use of the CaNDis web server's innovative network visualization to quickly suggest candidate proteins with potential new roles in co-occurrence of diseases.

## Funding

This work was supported by the Slovenian Research Agency [P2-0103, N2-0078 and N1-0142]. The first author (B.Š.) acknowledges support under the junior researcher programme.

*Conflict of Interest*: none declared.

## Supplementary Material

btaa762_Supplementary_Data
