# Supplementary material for: CaNDis: a web server for investigation of causal relationships between diseases, drugs and drug targets
Source: Bioinformatics. 2020 Sep 1;37(6):885–7. doi: 10.1093/bioinformatics/btaa762 (PMC8098028; doi:10.1093/bioinformatics/btaa762)
Supplement: btaa762_Supplementary_Data [file btaa762_supplementary_data.zip › supplementary-information-minor-rev-final.pdf]

Supplementary Information

CaNDis: A web server for investigation of causal relationships between diseases, drugs, and drug targets

Blaž Škrlj[1,2], Nika Eržen[1], Nada Lavrač[1,2], Tanja Kunej[3], Janez Konc[4]

[1]Department of knowledge technologies, Jožef Stefan Institute, Jamova 39, SI-1000 Ljubljana, Slovenia

[2]Jožef Stefan International Postgraduate School, Jamova 39, SI-1000 Ljubljana, Slovenia

[3]Biotechnical faculty, University of Ljubljana, Jamnikarjeva 101, SI-1000 Ljubljana, Slovenia

[4]Theory Department, National Institute of Chemistry, Hajdrihova 19, SI-1000 Ljubljana, Slovenia

Software aspects and scaling. The CaNDis web server is implemented in Node.js with the heterogeneous causal network and the disease-disease network persistent in RAM enabling fast queries for multiple diseases at once. The front-end is based on a fast WebGL-based interactive 3D network viewer available at https://github.com/vasturiano/3d-force-graph optimized to visualize metabolic pathways at the interface of the selected diseases. This viewer enables rapid and intuitive exploration of various interactions, e.g., disease-disease interactions. The data format of the network follows the Biological Expression Language (BEL) and can thus be easily extended with new heterogeneous data. The web server also provides various network statistics that enable the users to navigate and prioritize diseases to examine, including the global PageRank centralities, average degrees, and the number of edges segregated based on their types. The PageRank centralities are computed using the PageRank algorithm (Page *et al.*, 1999) with the default hyperparameter settings (damping factor of 0.85, uniform prior). Edge evidence score equals to the number of reported experiments that confirm a given interaction. The potential biomarker interactions are characterized by at least one of the partners being linked to either of the selected diseases.

Comparison with other tools. CaNDis web server is designed to answer the question which diseases (two or more) are related to each other due to the overlapping of the underlying interaction networks. It enables an intuitive exploration of the disease-disease network as well as the causal interaction networks. In contrast, the DISNOR web resource allows the causal interaction network for a single disease to be explored (Lo Surdo *et al.*, 2018). CaNDis also contains information on verified drugs, which is one of the most important innovations and provides insights into potentially interesting drug-drug interactions. In addition, CaNDis also provides insights into RNA-protein interactions, even though these are in minority compared to other interaction types. CaNDis thus offers a complementary service to other tools (Lo Surdo *et al.*, 2018), and focuses on the intuitive three-dimensional exploration of known causal interactions and diseases.

The CaNDis network. The CaNDis web server integrates the following databases into a heterogeneous causal network:

1. SIGNOR 2.0, curated protein-protein and other causal interactions (Licata et al., 2020);

2. CBN, causal interactions between various biological entities, including proteins, transcripts and genes (Boué et al., 2015);

3. PRD, interactions between RNA molecules and proteins (Fujimori et al., 2012);

4. DisGeNet, associations between proteins and existing diseases (Piñero et al., 2020);

5. DGIdb, interactions between FDA approved drugs and genes (Cotto et al., 2018).

The statistics of the CaNDis causal network and comparison to the underlying networks is in Table S1, and is also available under the “Data” section on the web server (Fig. S1). The disease-disease network consists of 5,648 nodes and 1,429,213 edges.

Table S1. The coverage of the CaNDis causal network (pathways network) compared with other causal biological network resources.

	CaNDis	SIGNOR 2.0*	CBN
Nodes	4,937	4,234	1,731
Edges	14,036	11,406	3,723
Node types	20	10	18
Edge types	16	11	4

*We included the SIGNOR 2.0 entries for *Homo sapiens* causal interactions that we could annotate with the adopted BEL formalism. The entries were also required to have a corresponding UniProtKB annotation and PubMed ID, to ensure high quality causal interactions; the interactions that differed in their PubMed IDs only, were merged.

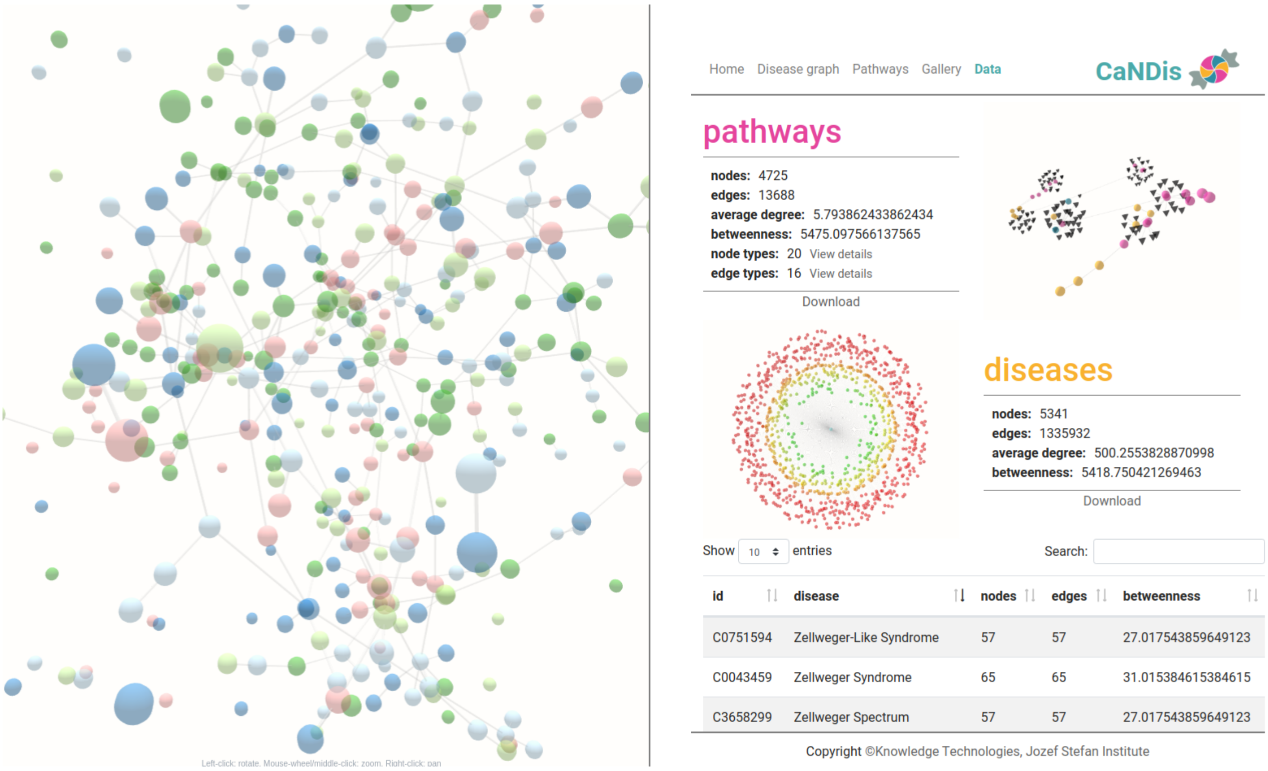

Fig. S1. Overview of the CaNDis network statistics. The "pathways" section contains the summary of the molecular graph at the level of proteins, transcripts, RNA molecules and drugs. The "diseases" section contains the induced disease-disease network and its key properties. The number of node and edge types can also be inspected for the "pathways" networks. At the bottom of the "Data" section, a tabular overview of individual disease networks is given. The "stats.json", an object containing the tabular summary in Json format, can be downloaded from the web server.

The CaNDis causal network consists of 120 connected components, whereas the corresponding disease-disease network is a single connected component. The node degree distributions of the causal and the disease-disease networks are shown in Figs. S2 and S3. The causal network's degree distribution most likely follows the power law or log-normal distribution as determined by fitting several alternative distributions to the network data (Fig. S2) (Alstott *et al.*, 2014).

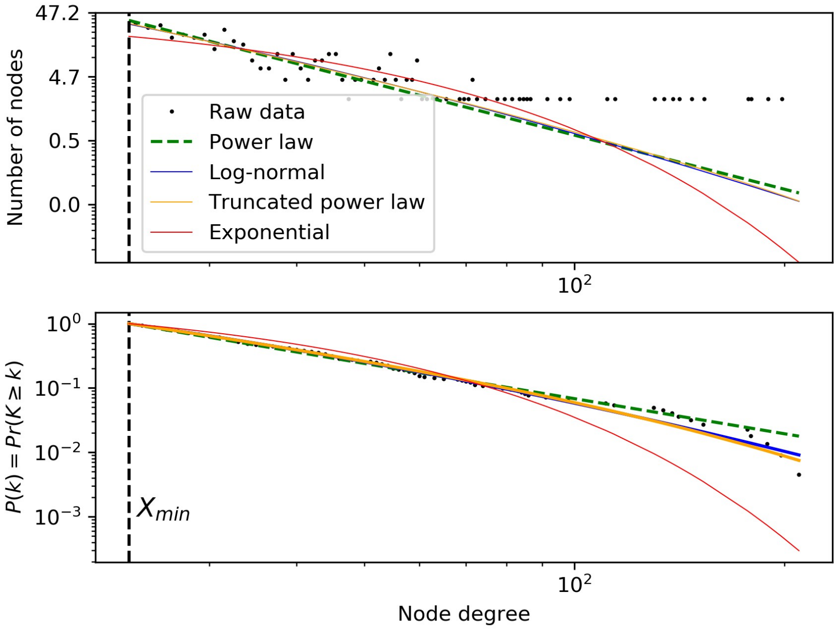

Fig. S2. Alternative distribution fits to the node degree distribution of the CaNDis causal network. Above: node degree counts; below: cumulative node degree counts.

On the other hand, the disease-disease network is much denser and does not follow any of the heavy-tailed distributions nor the non-heavy tailed exponential distribution (Fig. S3).

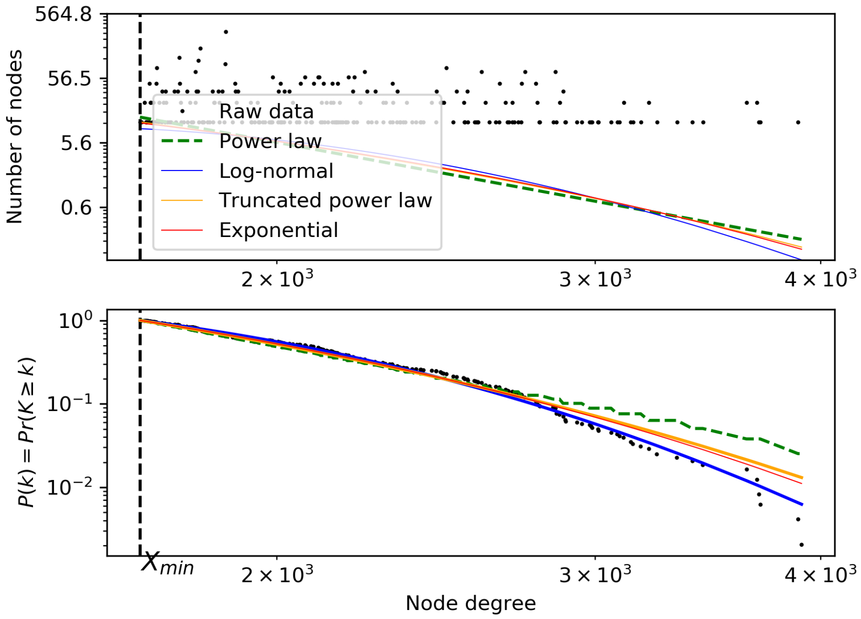

Fig. S3. Alternative distribution fits to the node degree distribution of the CaNDis disease-disease network. Above: node degree counts; below: cumulative node degree counts.

Use in drug-drug interactions. To show how the CaNDis web server enables finding proteins involved in drug-drug interactions, we look at the drugs erythromycin and verapamil. If these drugs are taken together, a potentially serious interaction occurs in which erythromycin enhances the effect of verapamil (Dakhel and Jamali, 2006). With CaNDis we observe that both erythromycin and verapamil interact with the interleukin 1 beta protein (gene name: *IL1B*), which is an important mediator of the inflammatory response (Fig. S4). This interaction is confirmed experimentally for both drugs individually (Takeshita *et al.*, 1989; Lamperi *et al.*, 1988), and there is also evidence that the drug-drug interaction may be related to altered interleukin levels (Sirmagul *et al.*, 2006; Kaeser *et al.*, 1998). The visualization of drug-protein interactions by CaNDis therefore enabled us to efficiently propose the interleukin 1 beta as a protein with a possible role in this known drug-drug interaction. Additional use cases are provided in the "Gallery" section of the CaNDis web server.

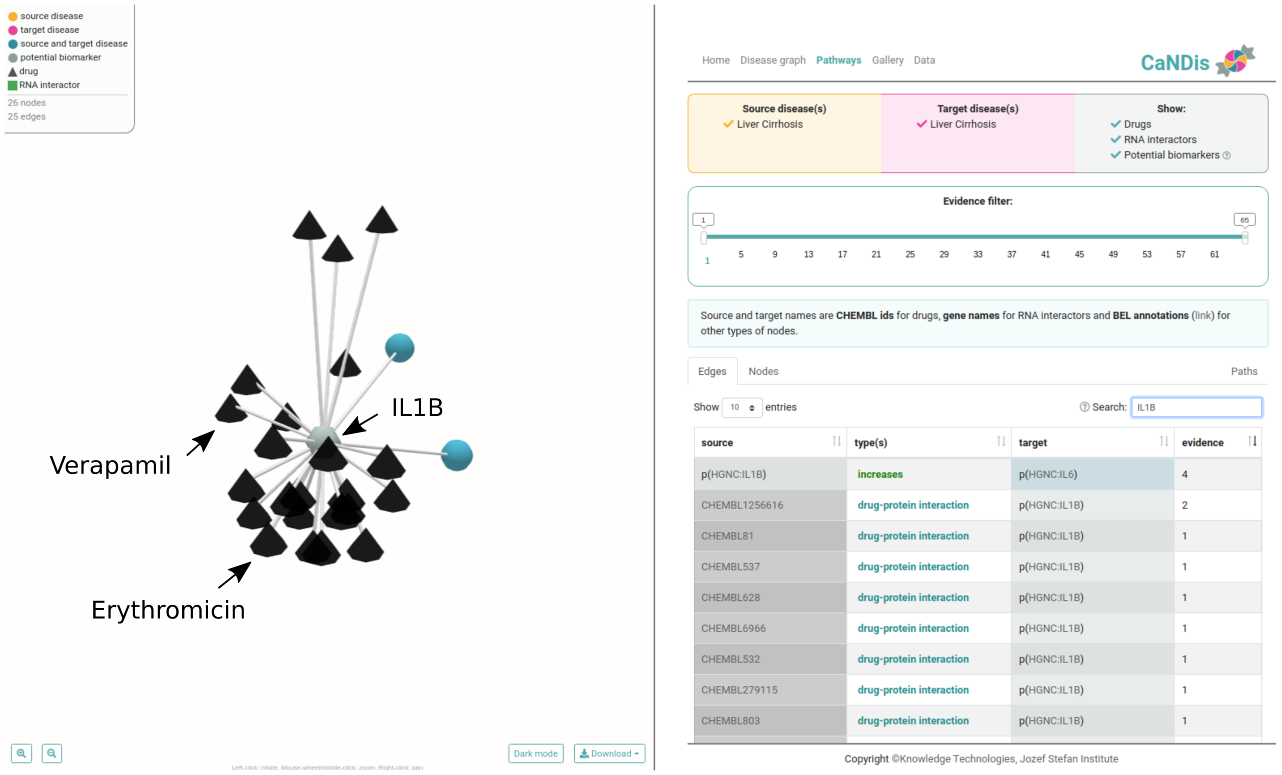

Fig. S4. CaNDis offers direct research into interactions between drugs. Left: the drugs verapamil and erythromycin bind to interleukin 1 beta (*IL1B*), which positions this protein as a candidate for a role in this drug-drug interaction. Right: tabular drug-protein interactions corresponding to the network on the left.

Disease-disease network construction. We have developed a new disease-disease network, which is available on the CaNDis web server (Fig. S5). In this network, a pair of diseases is connected if the diseases share a degree of similarity represented by a similarity score. This is the sum of the connections between node (gene) pairs where the first node belongs exclusively to the first disease and the second exclusively to the second disease and the connections between nodes that are

common to both diseases. This disease-disease similarity score represents the similarity of two diseases and if it equals zero, a connection is omitted.

The DisGeNET-based disease-gene associations do not necessarily imply causal relationships between a gene and a disease. Each such association is extracted from a publication, but the causality is often not determined. We have used these non-causal disease gene associations to annotate the CaNDis causal network composed from other databases (Boué *et al.*, 2015; Licata *et al.*, 2020; Fujimori *et al.*, 2012; Cotto *et al.*, 2018). This allows the user to explore the causal interaction network with superimposed additional information about diseases. To our knowledge this is not yet available in other tools.

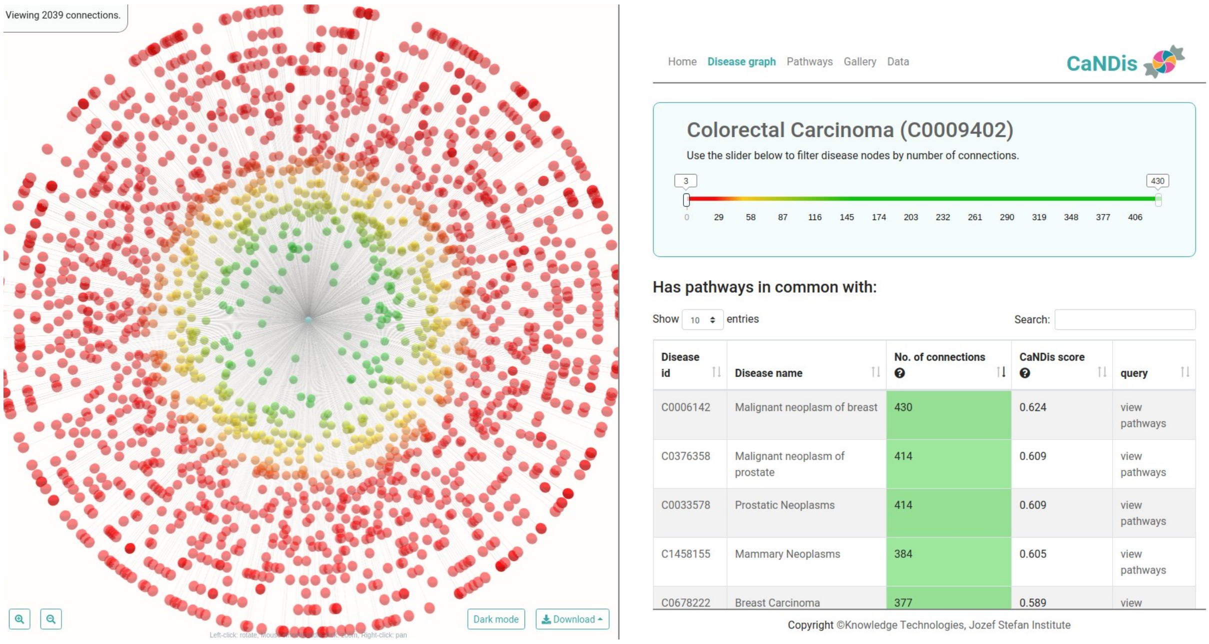

Fig. S5. A network of disease-disease interactions for the colorectal carcinoma. Left: diseases that are related through their common interacting molecules to colorectal carcinoma (central node) shown as a 3D network projection. Right: tabular view of the same network predicted by CaNDis. The diseases are ranked by the similarity score to the colorectal carcinoma. The edges can be queried and filtered. By clicking on “view pathways” button, a 3D visualization of interacting molecules, annotated with a given disease pair, is shown.

Normalized disease-disease similarity score. To rank diseases by similarity we introduce the normalized CaNDis disease-disease similarity score. This score takes into account the interactions that can occur randomly in the absence of a true association between diseases. It is based on the standard score introduced in the section “Disease-disease network construction” and is defined as follows. Let s(a,b) represent the standard score that corresponds to the number of edges linking a given pair of diseases a and b; let avg(a) and avg(b) represent the average scores calculated as an average of all possible standard scores for each of the diseases a and b to their corresponding connected diseases. The normalized CaNDis score is then calculated as CaNDisScore(a,b) = norm(s(a,b)/max(avg(a),avg(b))) where the norm is a standard normalization function that truncates

the score values to the interval [0,1]. This normalized score discriminates against disease pairs that are well connected, allowing a more balanced view of the disease-disease landscape.

References

Alstott,J. *et al.* (2014) powerlaw: A Python Package for Analysis of Heavy-Tailed Distributions. *PLOS ONE*, **9**, e85777.

Boué,S. *et al.* (2015) Causal biological network database: a comprehensive platform of causal biological network models focused on the pulmonary and vascular systems. *Database (Oxford)*, **2015**.

Cotto,K.C. *et al.* (2018) DGIdb 3.0: a redesign and expansion of the drug–gene interaction database. *Nucleic Acids Res.*, **46**, D1068–D1073.

Dakhel,Y. and Jamali,F. (2006) Erythromycin potentiates PR interval prolonging effect of verapamil in the rat: A pharmacodynamic drug interaction. *Toxicol. Appl. Pharmacol.*, **214**, 24–29.

Fujimori,S. *et al.* (2012) PRD: A protein–RNA interaction database. *Bioinformation*, **8**, 729–730.

Kaeser,Y.A. *et al.* (1998) Severe hypotension and bradycardia associated with verapamil and clarithromycin. *Am. J. Health. Syst. Pharm.*, **55**, 2417–2418.

Lamperi,S. *et al.* (1988) Intraperitoneal verapamil therapy in CAPD patients with peritoneal hypopermeability. Effects on ultrafiltration. - Abstract - Europe PMC. *ASAIO Trans.*, **34**, 425–428.

Licata,L. *et al.* (2020) SIGNOR 2.0, the SIGnaling Network Open Resource 2.0: 2019 update. *Nucleic Acids Res.*, **48**, D504–D510.

Lo Surdo,P. *et al.* (2018) DISNOR: a disease network open resource. *Nucleic Acids Res.*, **46**, D527–D534.

Page,L. *et al.* (1999) The PageRank Citation Ranking: Bringing Order to the Web. Technical Report. Stanford InfoLab.

Piñero,J. *et al.* (2020) The DisGeNET knowledge platform for disease genomics: 2019 update. *Nucleic Acids Res.*, **48**, D845–D855.

Sirmagul,B. *et al.* (2006) Effects of verapamil and nifedipine on different parameters in lipopolysaccharide-induced septic shock. *Heart Vessels*, **21**, 162–168.

Takeshita,K. *et al.* (1989) Immunological and anti-inflammatory effects of clarithromycin: inhibition of interleukin 1 production of murine peritoneal macrophages. *Drugs Exp. Clin. Res.*, **15**, 527–533.
